# Association of Periodontal Red Complex Bacteria With the Incidence of Gastrointestinal Cancers: A Systematic Review and Meta-Analysis

**DOI:** 10.7759/cureus.59251

**Published:** 2024-04-29

**Authors:** Sriram Kaliamoorthy, Sugantha Priya Sayeeram, N Gowdhaman, Merlin Jayaraj, B Radhika, Sugirtha Chellapandi, Agila Elumalai, Sai P Archana, Kanmani Raju, Santosh Palla

**Affiliations:** 1 Department of Dentistry, Vinayaka Missions Medical College and Hospital, Vinayaka Missions Research Foundation, Karaikal, IND; 2 Department of Prosthodontics, Government Dental College and Hospital, The Tamil Nadu Dr. MGR Medical University, Pudukkottai, IND; 3 Departmentof Physiology, Dhanalakshmi Srinivasan Medical College and Hospital, The Tamil Nadu Dr. MGR Medical University, Perambalur, IND; 4 Department of Oral and Maxillofacial Pathology, Chettinad Dental College and Research Institute, The Tamil Nadu Dr. MGR Medical University, Chennai, IND; 5 Department of Periodontics, Chettinad Dental College and Research Institute, The Tamil Nadu Dr. MGR Medical University, Chennai, IND; 6 Department of Oral Medicine and Radiology, Chettinad Dental College and Research Institute, The Tamil Nadu Dr. MGR Medical University, Chennai, IND; 7 Department of Oral Medicine and Radiology, Sun Dental Care, Chennai, IND

**Keywords:** p.gingivalis, red complex microbes, gastrointestinal cancers, periodontitis, oral cancer

## Abstract

*Porphyromonas gingivalis* is the primary microbe in the “periodontal red complex” bacteria (PRCB) along with *Tannerella forsythia* and *Treponema denticola*, which are linked to periodontal disease (PD). These pathogens are also implicated in various systemic disorders, but their association with the incidence of gastrointestinal (GI) cancer is less explored. A systematic review followed by a meta-analysis was conducted as per standard guidelines (Preferred Reporting Items for Systematic Reviews and Meta-Analysis (PRISMA) 2022) to find this association between GI cancers and PRCB after a literature search for full-text papers in the English language (between 2010 and 2023) in databases (Cochrane Library, PubMed, and Web of Science) with suitable keywords using the Boolean search strategy. Data extraction involved titles, abstracts, and full texts retrieved and scored by the modified Newcastle-Ottawa Scale. The data were analyzed by the Review Manager (RevMan 5.2, Cochrane Collaboration, Denmark). Standard Cochran Q test and I^2^ statistics (for heterogeneity) and a random effects model (pooled OR with 95% CI) were applied to report results. *P. gingivalis* among the PRCB was linked to GI cancers (OR: 2.16; 95% CI: 1.34-3.47). *T. forsythia* and *T. denticola* did not show meaningful associations as per existing evidence for GI cancers.

## Introduction and background

Gastrointestinal (GI) cancers represent the varied types of cancers occurring in the digestive system, which include esophageal, gastric, colorectal, pancreatic, and hepatocellular cancers [1]. The global incidence is alarming, with 35% of all deaths caused by cancers. The incidence of GI cancers accounted for 26% of all types of cancers worldwide. The frequency of GI cancers is double in men compared to women. It was reported that the distribution of certain variants of GI cancers is explicit in different regions of the world. Gastric, liver, and esophageal variants were common in Asia [2]. Alcohol and tobacco consumption, poor lifestyle (and related disorders such as obesity and overweight), *Helicobacter pylori* infection, and gut dysbiosis are known risk factors for GI cancers [3]. It was reported that the global mortality rate due to GI cancers in 2008 was 39.4 cases per 100,000 individuals, while the incidence rate was 54.9 cases per 100,000. Colorectal and stomach cancers were ranked as the third and fourth most common cancers, respectively. The highest mortality rate was observed among those with stomach cancer (26.1%) of all GI cancers [4].

Oral dysbiosis, or altered oral microbiota, was recently linked to have a significantly positive association with GI cancers (esophageal, pancreatic, and colorectal cancer (CRC)). Dysbiosis of the oral microbiota is caused by two major oral pathologies: dental caries and periodontitis or periodontal disease (PD). Increased colonization of subgingival gram-negative anaerobic bacteria results in deep pockets, loss of clinical attachment, and bleeding on probing (BOP), all corresponding to PD [5,6]. The advancement of PD leads to a transition of oral microorganisms toward the “periodontal red complex” bacteria (PRCB) (*Porphyromonas gingivalis*, *Tannerella forsythia*, and *Treponema denticola*), which are known to contribute to the carcinogenesis of GI cancers. Altered oral microbes with periodontal pathogens like *T. forsythia*, *Streptococcus anginosus*, *Prevotella intermedia*, and *P. ginigivalis* demonstrated a high risk of esophageal adenocarcinoma (EAC). *Fusobacterium nucleatum* and *T. denticola* were linked to intensifying the risk of CRC, while *P. gingivalis* contributed to the pathogenesis of pancreatic cancer (PC) [7]. Overall, PRCB was concomitant with the risk of developing different types of GI cancers [8].

PRCB presence is linked to many other systemic diseases or conditions, i.e., from liver cirrhosis to chronic kidney disease [9,10]. *P. gingivalis* was reported to promote the occurrence and to worsen the prognosis of esophageal cancer (EC) [11]. *T. forsythia* and *P. gingivalis* are known to induce a systemic inflammatory response, TNF-alpha pathway activation, and in the generation of reactive oxygen species (ROS) as a part of their GI carcinogenesis [12]. Oral microbes inhibit the host immune response and alter the regular inflammatory signaling pathways, leading to an eventful longstanding chronic PD. Periodontal pathogens are also identified as risk factors for atherosclerosis, diabetes, chronic obstructive pulmonary disease, and rheumatoid arthritis, all of which seem to have chronic inflammation and immune alteration in their basic pathogenesis [7,13].

The oral cavity harbors an intricate ecosystem of microbiota, playing a crucial role in pathogen resistance, homeostasis maintenance, and immune system modulation. Growing evidence points to the potential implication of oral microbiota in inducing and promoting GI cancers. This study aimed to identify the role of PRCB in the incidence of various GI cancers, which is a topic less studied in dental literature.

## Review

Methods

Research Question

The review was conducted as per the standards of the Preferred Reporting Items for Systematic Reviews and Meta-Analysis (PRISMA) guidelines [14]. The research question (as per Participants, Exposure, Control, and Outcomes (PECO)) principle was the following: "Is PRCB infection associated with GI cancers?" P (Participants): subjects with periodontitis associated with one or more PRCB; E (Exposure): PRCB; C (Control): controls (subjects without PD); and O (Outcome): incidence of reported GI cancers.

Selection Process

The prospective or retrospective studies that compared patients with PRCB infection vs. PRCB uninfected cases of GI cancer, studies that had standards for the diagnosis of cancer (confirmed by histopathological examination) and PD (diagnosed by clinical diagnostic criteria), those in line with the current research question, those studies carried out in adults of either gender who were 18 years of age (or above), and those studies that analyzed the cancer incidence rates were eligible. Studies that defined GI cancers/PD without a standard clinical diagnosis did not measure defined outcomes, were published in languages other than English, or were narrative reviews or animal studies were omitted.

Search Strategy

The search strategy involved the use of “keywords or near matches” (including the Medical Subject Headings (MeSH) terms) separated by the bullion operator (Boolean search strategy) "AND or "OR" as per the need. This may be described as (((cancer [Title/Abstract]) OR malignancy [Title/Abstract]) OR carcinoma [Title/Abstract]) AND (((*Porphyromonas gingivalis* [Title/Abstract]) OR *Tannerella forsythia* [Title/Abstract]) OR *Treponema denticola* [Title/Abstract]). MEDLINE/PUBMED and Cochrane Library databases were searched from January 2010 to June 31, 2023. The full texts of eligible papers were assessed by the first four authors (A1-A4), with conflicts (if any) being resolved by the next author (A5). The remaining authors (A6-A9) were acting as second tire reviewers, and the last acted as the guarantor (A10).

Data Extraction

Authors (A7, A8, and A9) independently extracted meta-data about the year of publication, isolation method, names of PRCB, sample size, type of GI cancer, and any other significant findings. The studies with relevant titles were collected as per the search strategy, and abstracts were read manually by these authors, if found suitable with defined eligibility, the full texts were obtained and read. The enrolled studies were checked for their value as per the PRISMA checklists by one author (A10).

Data Items

The data of the authors, year of publication, isolated PRCB species, the organs affected under GI cancers, the sample size of cases, and the controls were metadata retrieved after screening full texts of eligible studies. Also, the diagnostic methods used and principal outcomes reported from each study were retrieved along with the said meta-data of selected studies.

Effect Measures

The data were collected and analyzed by Review Manager 5.2 (RevMan 5.2, Cochrane Collaboration, Denmark). The heterogeneity between the included studies was declined by the standard Cochran Q test and I^2^ statistics, where a value of I^2^ statistics >50%, and a P < 0.1 was taken for significant heterogeneity. The pooled OR and 95% CI were calculated by a random effects model in these cases. A fixed effects model was applied generally when the heterogeneity was not significantly detected. A mean sample size was taken as a boundary when studies had gross differences or small and large sample sizes.

Quality and Reporting Risk of Bias

The modified Newcastle-Ottawa Scale was used for scoring the quality of included studies, keeping in mind factors such as selection, comparability, and outcomes [15]. The studies included after final scoring are as per the final review by a team of four authors (A5-A9). Also, two reviewers analyzed the risk of bias (ROB) using the Egger and Begg regression intercept tests. The STATA 12 (StataCorp LLC, College Station, Texas, USA), wherein a P < 0.05 (two-tailed), was taken for significance.

Results

Study Characteristics and Results of Syntheses

**Figure 1:**
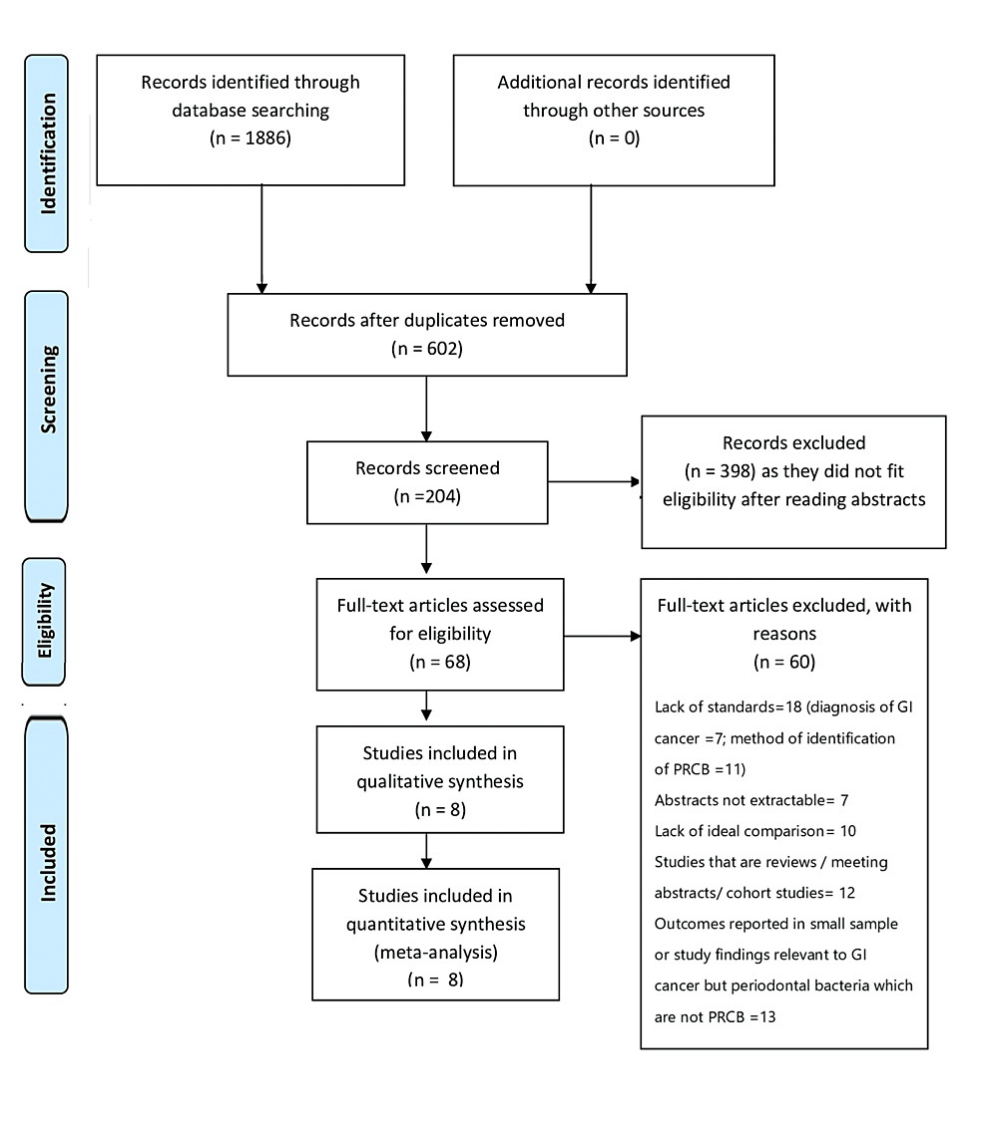
PRISMA flow diagram GI, gastrointestinal; PRCB, periodontal red-complex bacteria; PRISMA, Preferred Reporting Items for Systematic Reviews and Meta-Analysis

In total, we had a hit of 1,886 search findings from all the mentioned databases within a set timeframe. No further leads were obtained from other sources, such as unpublished data or pilot study results, after verification with authors via personal emails or social platforms (ResearchGate). Of these, 602 were obtained after removal of duplicates, and 204 after excluding (n = 398) reading the abstracts. The authors deemed 68 full papers eligible for review after excluding 60. Finally, eight studies [16-23] were considered for qualitative and quantitative analyses. The process and reasons for elimination at each step are shown in the PRISMA flowchart (Figure 1).

Most of the studies had a good sample size (range: 80-821). Of the eight studies, the majority, i.e., Peters et al. [16], Chang et al., and Yuan et al. [23] have studies have analyzed PRCB mostly from patients with EC; two studies each studied PC and CRC. One study by Sun et al. [19] studied precancerous lesions, which was also taken into account here. For the isolation of PRCB samples, 16S rRNA gene sequences from mouthwash were a common method, followed by the use of saliva/blood-based detection by quantitative polymerase chain reaction (qPCR)/immunoblot assays. Kerdreux et al. [22], in cases of CRC, studied the mucosal and fecal samples, while Chang et al. [21] studied cancer tissue specimens directly for the bacteria. All the authors have explored links between GI cancer and *P. gingivalis*. *T. forsythia* was next commonly researched, while *T. denticola* was least explored. Peters et al. [16], Sun et al. [19], and Yang et al. [20] have studied all PRCB and other periodontal pathogens.

*P. gingivalis* was linked to all types of GI cancers, namely, esophageal squamous cell carcinoma (ESCC)/EAC (EC), PC, and CRC. *T. forsythia* was linked to EAC/EC but not studied or found linked to PC. *T. denticola* was the least studied PRCB, but as per one study, it was reported to be associated with poor hygiene practices in CRC. For precancers or gastric precancerous lesions (GPCL), surprisingly, *P. gingivalis* was found to be associated, but *T. forsythia* and *T. denticola* were significantly found to be associated. The details of each study and their quality assessments are shown in Table 1, while their respective quality assessments are in Table 2.

**Table 1:** Characteristics of included studies in the systematic review CAL, clinical attachment loss; CRC, colorectal cancer; EAC, esophageal adenocarcinoma; EC, esophageal cancer; ESCC, esophageal squamous cell carcinoma; GPCL, gastric precancerous lesions; IHC, immunohistochemistry; MSI, micro-satellite; PC, pancreatic cancer; PCR, polymerase chain reaction; PRCB, “periodontal red complex” bacteria; *P. gingivalis*, *Porphyromonas gingivalis*; p, significance; *T. denticola*, *Treponema denticola*; *T. forsythia*, *Tannerella forsythia* *P < 0.01 is taken for significance in all instances.

Authors	Year	Study design	Mean age/age range/age groups	Age	PRCB	Type of GI cancer	Cases	Controls	Diagnostic method	Outcomes reported	Confounders (if any, adjusted for)
Peters et al. (n = 316) ^[16]^	2017	Population-based nested case-control study	Age range: 50-74		*P. gingivalis*, *T. forsythia*, and *T. denticola*	EC (EAC/ESCC)	106	210	16S rRNA gene sequences from mouth rinse samples	*T. forsythia* was linked with a higher risk of EAC [1.21 (1.01, 1.46)]. *P. gingivalis* was linked with a higher risk of ESCC [1.30 (0.96, 1.77)]. *T. denticola* had no significant associations.	BMI, smoking, and alcohol.
Fan et al. (n = 732) ^[17]^	2018	Population-based nested case-control study	Age range: 50-74		*P. gingivalis* and *T. forsythia*	PC	361	371	16S rRNA gene sequences from mouth rinse samples	*P. gingivalis* was associated with a high risk of cancer (adjusted OR =1.60 (1.15-2.22)). *T. denticola* and *T. forsythia* had no significant associations.	age, sex, race, BMI, smoking status, alcohol status, and history of diabetes.
Michaud et al. (n = 821) ^[18]^	2013	Cohort study	Age range: 35-70 years		*P. gingivalis* and *T. forsythia*	PC	405	416	Immunoblot array on blood samples	High levels of antibodies against *P. gingivalis* implied a two-fold higher risk of PC as opposed to those with lower titers of bacteria (OR = 2.14 (1.05-4.36); >200 ng/mL vs. ≤200 ng/mL). *T. forsythia* was reported with no significant associations.	BMI, waist circumference, current and past tobacco smoking, and diabetes.
Sun et al. (n = 105) ^[19]^	2017	Case-control study	Mean age: 57.98 ± 9.0 years (range: 36.3-82.6)		*P. gingivalis*, *T. forsythia*, and *T. denticola*	GPCL	35	70	qPCR on saliva samples	Patients with GPCL experienced a higher prevalence of spiked *T. denticola* levels (P < 0.01) as opposed to controls. Spiked colonies of *T. forsythia* and *T. denticola*, decreased bacterial plaque diversity, and hygiene practice of "not flossing" were associated with an increased risk of GPCL (P = 0.022). No links with *P. gingivalis* were reported.	Race/ethnicity, gender, and age.
Yang et al. (n = 692) ^[20]^	2019	Nested case-control study	Age groups: 40-49; 50-59; 60-69; 70-79		*P. gingivalis*, *T. forsythia*, and *T. denticola*	CRC	231	461	16s rRNA gene sequences (mouthwash samples)	*T. denticola* (1.76(1.19–2.60)) was associated with an increased risk of CRC. No associations with *T. forsythia* and *P. gingivalis* were reported.	Smoking (in pack-years) and alcohol consumption
Chang et al. (n = 91) ^[21]^	2019	Observational study (three groups, cancer vs. precancer vs. normal)	Age groups: ≤60 and >60		P. gingivalis	ESCC	61	30	qPCR using a special oligonucleotide probe on cancer tissue samples	*P. gingivalis* was detected in 60.7% of OSCC tissues as opposed to 13.3% of normal tissues. *P. gingivalis* infection was linked to poor prognosis in patients with OSCC who had PD progression (tooth loss, deep pockets, or higher rates of CAL).	Not mentioned
Kerdreux et al. (n = 435) ^[22]^	2023	Case-control study	Age groups: ≤ 59; 60-69;70-79; ≥ 80)		P. gingivalis	CRC	247 (cohort 1); 128 + 38 (cohort 2)	89 (cohort 1); 61 (cohort 2)	Quantitative real-time PCR-based detection from mucosal and fecal samples	*P. gingivalis* was detected significantly in the feces of CRC patients compared to controls (P = 0.028). A positive association was found between the presence of *P. gingivalis* in feces and tumor tissue (P < 0.001).	Not mentioned
Yuan et al. (n = 80) ^[23]^	2017	Observational study (three groups, cancer vs. precancer vs. normal)	Not mentioned		P. gingivalis	EC	50	30	16S rDNA and qPCR amplification comparisons form cancerous tissues	*P. gingivalis* was frequently present in specimens of EC vs. non-cancerous portions (48% vs. 3%; χ^2^ =17.412; P < 0.001). *P. gingivalis* was preferentially present in specimens of dysplastic esophagus vs. non-dysplastic portions (40% vs. 20%, χ^2^ = 4.059; P = 0.044). *P. gingivalis* was significantly present in esophagus cancers (region with low acidity) > cardia/stomach cancers (regions with high acidity).	Not mentioned

**Table 2:** Quality assessments as per the modified Newcastle-Ottawa Scale for selected studies Newcastle-Ottawa Scale quality instrument is scored by awarding a point for each answer that is marked with an asterisk (*). The maximum total score is nine points, with 0-2 points indicating poor quality, 3-5 points indicating fair quality, and 6-9 points indicating good quality. The scores are given in column 3 as a, b, c, etc., corresponding to the respective criteria listed in column 2. Most of the included studies had low bias or were under the “fair quality” cadre.

Study design	Criteria for scoring	Peters et al.	Fan et al.	Michaud et al.	Sun et al.	Yang et al.	Chang et al.	Kerdreux et al.	Yuan et al.
Selection of just one star (*) given for each question	Is the case definition adequate? a) yes, with independent validation*; b) yes, record linkage or based on self-reports, no description; c) no description	a*	a*	a*	a*	a*	a*	a*	a*
	Representativeness of the cases: a) consecutive or obviously representative series of cases*; b) potential for selection biases or not stated	a*	a*	a*	a*	a*	a*	a*	a*
	Selection of controls: a) community controls* ; b) hospital controls; c) no description	b	a*	b	a	b	a	a	a
	Definition of controls: a) no history of the disease (endpoint)*; b) no description of the source	a*	a*	b	b	a*	b	b	b
Comparability: Up to two stars (*) given for each question	Comparability of cases and controls on the basis of the design or analysis: a) study controls for age*; b) study controls for the duration of hospitalization*	a*	b	a*	b*	a*	a*	b*	b*
Exposure: Up to one star (*) given for each question	Ascertainment of exposure: a) secure record (e.g., surgical records)*; b) structured interview blinded to case/control status; c) interview not blinded to case/control status; d) written self-report or medical record only; e) no description	a*	a*	a*	a*	a*	a*	a*	a*
	Same method of ascertainment for cases and controls: a) yes*; b) no	a*	a*	a*	a*	a*	a*	a*	a*
	Nonresponse rate: a) same rate for both groups*; b) nonrespondents described rate different and no designation; c) no description	a*	a*	a	c	a*	c	c	c
Score		8	7	6	7	8	6	7	7

The findings demonstrated that individuals who had an infection with *P. gingivalis* faced a 2.16 times greater GI cancer risk as opposed to those who were uninfected (OR = 22.02 (95% CI: 1.34-3.12); P = 0.001). *T. denticola* and *T. forsythia* did not have significant associations (Table 3).

**Figure 2:**
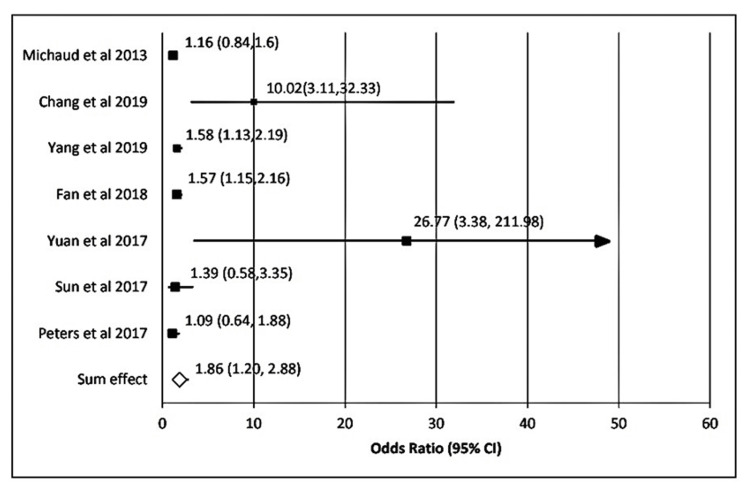
Forest plot showing the association of P. gingivalis with the incidence of gastrointestinal (GI) cancers Forest plot showing individual OR (black boxes) taken from selected studies (on the y-axis) and range (x-axis) for associating *P. gingivalis* with the incidence of GI cancers. The sum effect or pooled OR derived was OR; 95%CI, P) = [1.86(1.20,2.88) P = 0.001] (white diamond mark).

**Figure 3:**
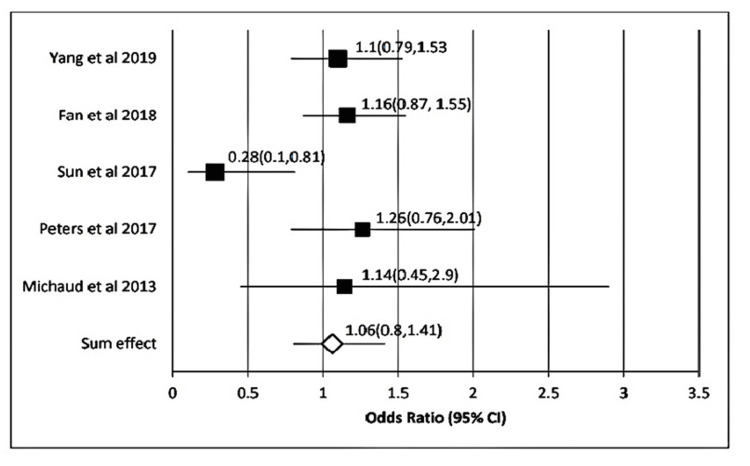
Forest plot showing the association of T. forsythia with the incidence of gastrointestinal (GI) cancers Forest plot showing individual OR (black boxes) taken from selected studies (on the y-axis) and range (x-axis) for associating *T. forsythia* with the incidence of GI cancers. The sum effect or pooled OR derived was OR; 95% CI, P) = [1.06 (95% CI, 0.8-1.44) P = 1.0] (white diamond mark).

**Figure 4:**
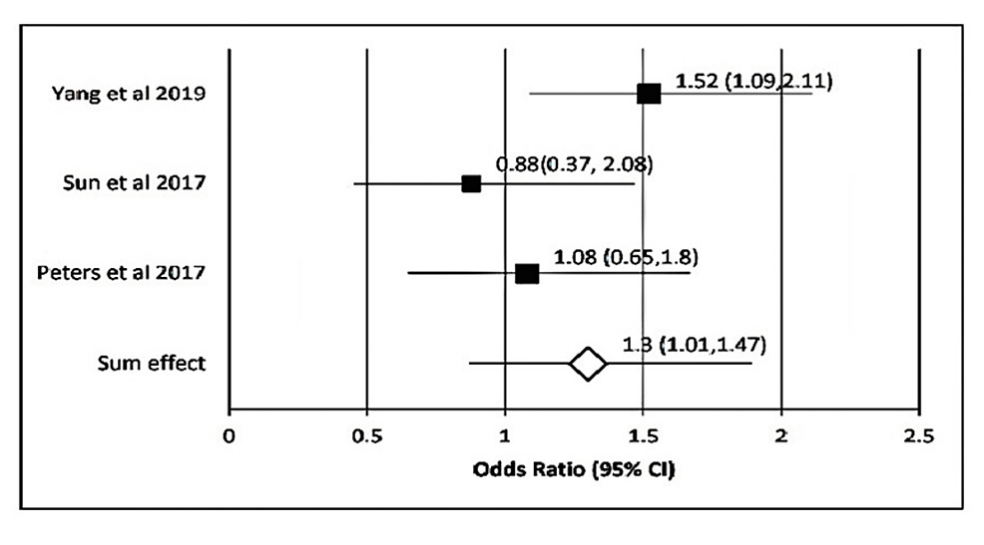
Forest plot showing the association of T. denticola with the incidence of gastrointestinal (GI) cancers Forest plot showing individual OR (black boxes) taken from selected studies (on the y-axis) and range (x-axis) for associating *T. denticola* with the incidence of GI cancers. The sum effect or pooled OR derived was [OR; 95%CI, P) = [1.31(0.97-1.71) P = 0.6] (white diamond mark).

In the case of *P. gingivalis*, the sum effect obtained was 1.86 (95% CI: 1.20-2.88). Heterogenicity calculated here showed τ^2^ = 0.28; χ^2^ = 36.41; *df* = 7; P < 0.00001; I^2^ = 82.0%. The test for overall effect was 3.47 (P = 0.001) (Figure 2). Likewise, for *T. forsythia*, the sum effect obtained was 1.06 (95% CI, 1.20-2.88). Heterogenicity calculated here showed τ2 = 0.04; χ^2^ = 6.7; *df* = 4 (P = 0.15) ; I^2^ = 40.0%. The test for overall effect was 0.42 (P = 0.66) (Figure 3). In the case of *T. denticola*, the sum effect obtained was 1.86 (95% CI: 1.20-2.88). Heterogenicity calculated here showed τ^2^ = 0.0; χ^2^ = 2.13; *df* = 7 (P = 0.35); I^2^ = 6.0%. The test for overall effect was 1.9 (P = 0.06) (Figure 4).

**Table 3:** Association of PRCB with the incidence of GI cancer PRCB, periodontal red complex bacteria; *P. gingivalis*, *Porphyromonas gingivalis*; *T. denticola*, *Treponema denticola*; *T. forsythia*, *Tannerella forsythia* *P < 0.01 is taken for statistical significance.

PRCB	Studies (incidence (cases/controls))	OR (95% CI)	df (P value)	I^2 ^(P value)	Overall Z (P values)
P. gingivalis	8 (1352/1632)	2.02 (1.34-3.12)	7(0.00005)	81%	3.01(0.01*)
T. forsythia	5 (409/626)	1.06 (0.8-1.44)	4(0.156)	80%	0(1.0)
T. denticola	3 (207/740)	1.31(0.97-1.71)	2(0.31)	6%	1.8(0.06)

Subgroup analysis (which included the total sample for each PRCB from all studies) also revealed similar results favoring *P. gingivalis* among the PRCB to be associated with cancer incidence (Table 4).

**Table 4:** Subgroup analysis for association of PRCB with the incidence of gastrointestinal (GI) cancer PRCB, periodontal red complex bacteria; *P. gingivalis*, *Porphyromonas gingivalis*; *T. denticola*, *Treponema denticola*; *T. forsythia*, *Tannerella forsythia* *P < 0.01 is taken for statistical significance.

PRCB	Studies (sample)	OR (95% CI)	Significance	I^2 ^(P values)
P. gingivalis	8 (n = 299)	1.86 (1.20-2.88)	0.005*	79% (P < 0.000)
T. forsythia	5 (n = 2,711)	1.06 (0.80-0.41)	0.67	67 41% (P = 0.15)
T. denticola	3(n = 1,113)	1.30 (0.99-1.72)	0.06	6% (P = 0.35)
All PRCB	24 (n = 10,656)	1.22 (1.01-1.47)	0.02	70% (P < 0.00001)

**Figure 5:**
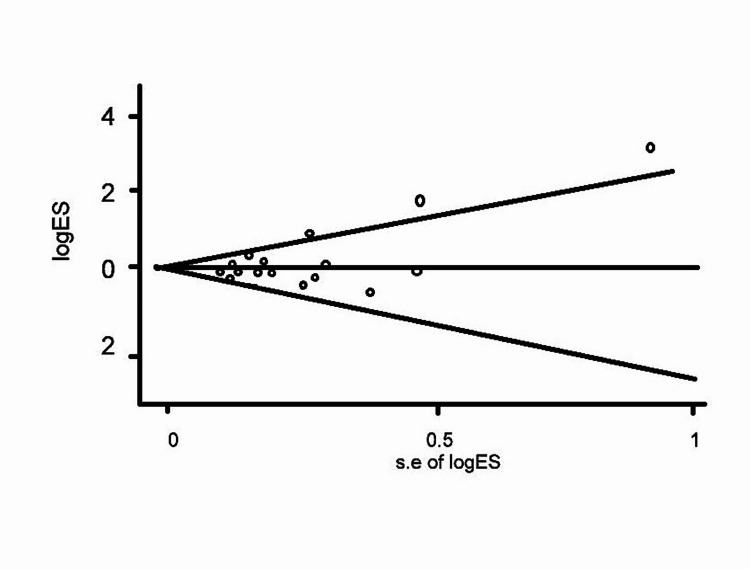
Assessments of risk of bias for studies/publications The Begg and Egger regression intercept test indicated no potential publication bias for the incidence of GI cancers (0.091). A two-tailed *P < 0.05 was considered statistically significant. GI, gastrointestinal

The ROB (Egger and Begg tests) with publications showed no potential publication bias for incidence (0.091) of GI cancers (Figure 5).

Discussion

The relationship between tumorigenesis and the activation of chronic inflammation is well-established [24]. *H. pylori* being associated with pathogenies of GI cancer is direct evidence of the fact that microbial infection can alter the inflammatory signaling pathway and aid in the propagation of carcinogenesis. The absence of timely intervention in such a case may lead to increased spread and, thus, poor prognosis for GI cancers [25]. Researchers have taken an interest in the linking between inflammation (caused by microbiota in PD) and the impact of the same on the onset and/or advancement of systemic diseases. These were proven true for many conditions ranging from Alzheimer's disease to cancers [26]. *P. gingivalis* of the PRCB is reported to promote oral cancers via ATR and NLRP3 inflammasome regulation or γδ T cell activation [27,28]. However, the association between these PRCB and the incidence of GI cancers needs to be studied as the oral cavity is an extension of the GI system or alimentary canal.

The current systematic review included eight studies that assessed the associated risk of one or all of the PRCB with the incidence of different types of GI cancers. The eight studies included in the review encompassed a total of 3,400 patients. Studies in recent decades have established a significant association between dysbiosis of the oral microbiota, especially colonization of red complex bacteria, and the incidence of GI cancers.

In the past, GI cancers were linked to smoking, alcohol consumption, obesity, and high cholesterol, all of which fall under the category of modifiable risk factors [29]. The more recent data from the past two to three decades have shown growing evidence that oral microbiota plays a significant role in GI cancers. Oral microbes possess a potential ability to trigger carcinogenic metabolism through various pathways in those who have traditional risk factors like smoking or alcohol consumption. This may be achieved by direct conversion of alcohol-/smoking-related carcinogens via oral microorganisms in both oral and GI cancers [30]. Oral microbiota with higher levels of subgingival periopathogens was found in patients with digestive system cancers. A study showed that gingival crevicular fluid (GCF) has a higher concentration of *T. forsythia*, *P. gingivalis*, and *T. denticola* in patients with gastric cancer diagnosed with PD [31].

*P. gingivalis* is one of the most studied PRCB and is shown to be concomitant in the pathogenesis of GI cancers. Tumorigenic markers like colony-stimulating factor 1 (CSF1), colon cancer-associated transcript 1 (CCAT1), and cellular invasion markers like MMP-9 were enhanced in *P. gingivalis* infected cells when patients with GI cancers and, more specifically, for CRC [32]. *P. gingivalis* was found to be positively associated with ESCC (also *P. gingivalis* IgG and IgA) and also increases the risk of PC by three times [33,34]. Available literature has elucidated the correlation between *P. gingivalis* and the development of ESCC through various methods. The salivary samples of the patients with ESCC were significantly high in *P. gingivalis* when compared to healthy individuals, as estimated using 16S rRNA gene sequencing. *P. gingivalis* is reported to promote the proliferation and metastasis of ESCC through NF-kB pathway activation [35]. Another study that analyzed tissue specimens from 100 ESCC patients detected *P. gingivalis* and Kgp enzymes in 61% of the samples through immunohistochemistry and qPCR [36]. These significant pieces of evidence suggest that *P. gingivalis* is associated with the development and progression of ESCC, aligning with the findings of the present review.

*P. gingivalis *was reported to be implicated in the tumorigenesis of PC by stimulating the genes (such as s100a8 or cxcl1/2) associated with neutrophil chemotaxis. This can be considered as a direct epigenetic mechanism in GI and pancreatic carcinogenesis [37]. A murine model demonstrated a higher genetic expression of Reg3g after stimulating the pancreas with *P. gingivalis* lipopolysaccharide, pointing to a possible association with PC [38]. An in vivo study showed that *P. gingivalis* can adhere, invade, and promote cell proliferation in host cells through MAPK and ERK pathways of colon cancer or CRC. It was also established that abundant P. *gingivalis* was observed in CRC tissue and fecal samples [39,40].

Mediators of inflammation (such as interleukin 1 and tumor necrosis factor) common to inflammation and carcinogenesis are triggered by *T. forsythia* to be produced from macrophages. *T. forsythia* was associated with EAC [41]. Likewise, the presence of *T. denticola* chymotrypsin-like protease (Td-CTLP) in various GI cancer specimens is documented. The IHC study demonstrated the presence of Td-CTLP in oropharyngeal SCC and other types of orodigestive cancer specimens (tongue, tonsils, and esophagus) [42,43]. These findings did not elucidate a direct association between *T. denticola* and the development of GI cancers, which needs further research. Yet another prospective study that explored the antibodies of PRCB about the incidence of cancer in 1,712 years of follow-up showed that *T. denticola* antibodies exhibited an inverse relationship to colon cancer (HR = 1.52) [44]. Put together, the existing data proposes that P. ginigivalis has a greater role in the development of GI cancers than the other two PRCBs, which is in line with the findings of the current study. The role of *T. denticola* and *T. forsythia* in the incidence of GI cancers requires further probing to determine proper incidences or associations, followed by understanding their molecular mechanisms. Also, we found that most studies done were on *P. gingivalis* only and not the other PRCB.

The limitations of the study lie in the selection of recent evidence only, which may have underrepresented papers on *T. denticola*. Similarly, there was substantial overall heterogeneity, necessitating the use of random effects models for the analysis. Another limitation of the study is the differences in the medium from which PRCB was extracted. Mouth rinse samples (studies on patients with PC, OC, or CRC) followed by blood, saliva, feces, and direct isolation from tissue were done in these studies. Each medium used is a known standard for studying that specific GI cancer (e.g., fecal samples for CRC), which led us to include the studies. However, the variations due to these differing samples used for PRCB extraction were not evaluated for their effect on the study outcome. The future directions may include studying more periodontal bacteria from populations of different ethnicities and adjusting for all described confounders with implications for GI cancers.

## Conclusions

The study showed that individuals infected with *P. gingivalis* have a raised risk of GI cancer by 2.16 times as opposed to those who are uninfected. *T. denticola* and *T. forsythia* did not show significant such associations. Oral prophylaxis, isolation of PRCB in those with chronic PD, and identification of reduced *P. gingivalis* loads post-periodontal therapy may have significant implications for patients with GI cancers.

## References

[1] Pourhoseingholi MA, Vahedi M, Baghestani AR (2015). Burden of gastrointestinal cancer in Asia; an overview. Gastroenterol Hepatol Bed Bench.

[2] Arnold M, Abnet CC, Neale RE, Vignat J, Giovannucci EL, McGlynn KA, Bray F (2020). Global burden of 5 major types of gastrointestinal cancer. Gastroenterology.

[3] Jardim SR, de Souza LM, de Souza HS (2023). The rise of gastrointestinal cancers as a global phenomenon: unhealthy behavior or progress?. Int J Environ Res Public Health.

[4] Hu QD, Zhang Q, Chen W, Bai XL, Liang TB (2013). Human development index is associated with mortality-to-incidence ratios of gastrointestinal cancers. World J Gastroenterol.

[5] Thomas C, Minty M, Vinel A (2021). Oral microbiota: a major player in the diagnosis of systemic diseases. Diagnostics (Basel).

[6] Mohanty R, Asopa SJ, Joseph MD, Singh B, Rajguru JP, Saidath K, Sharma U (2019). Red complex: polymicrobial conglomerate in oral flora: a review. J Family Med Prim Care.

[7] Zhang Y, Niu Q, Fan W, Huang F, He H (2019). Oral microbiota and gastrointestinal cancer. Onco Targets Ther.

[8] Chen Y, Chen X, Yu H, Zhou H, Xu S (2019). Oral microbiota as promising diagnostic biomarkers for gastrointestinal cancer: a systematic review. Onco Targets Ther.

[9] Nagao Y, Tanigawa T (2019). Red complex periodontal pathogens are risk factors for liver cirrhosis. Biomed Rep.

[10] Mahendra J, Palathingal P, Mahendra L (2022). Impact of red complex bacteria and TNF-α levels on the diabetic and renal status of chronic kidney disease patients in the presence and absence of periodontitis. Biology (Basel).

[11] Kong J, Liu Y, Qian M, Xing L, Gao S (2023). The relationship between Porphyromonas gingivalis and oesophageal squamous cell carcinoma: a literature review. Epidemiol Infect.

[12] Malinowski B, Węsierska A, Zalewska K (2019). The role of Tannerella forsythia and Porphyromonas gingivalis in pathogenesis of esophageal cancer. Infect Agent Cancer.

[13] Bourgeois D, Inquimbert C, Ottolenghi L, Carrouel F (2019). Periodontal pathogens as risk factors of cardiovascular diseases, diabetes, rheumatoid arthritis, cancer, and chronic obstructive pulmonary disease-is there cause for consideration?. Microorganisms.

[14] Liberati A, Altman DG, Tetzlaff J (2009). The PRISMA statement for reporting systematic reviews and meta-analyses of studies that evaluate health care interventions: explanation and elaboration. J Clin Epidemiol.

[15] Gierisch JM, Beadles C, Shapiro A (2014). Health Disparities in Quality Indicators of Healthcare Among Adults with Mental Illness [Internet]. Health disparities in quality indicators of healthcare among adults with mental illness [internet]. Washington (DC): Department of veterans affairs (US).

[16] Peters BA, Wu J, Pei Z (2017). Oral microbiome composition reflects prospective risk for esophageal cancers. Cancer Res.

[17] Fan X, Alekseyenko AV, Wu J (2018). Human oral microbiome and prospective risk for pancreatic cancer: a population-based nested case-control study. Gut.

[18] Michaud DS, Izard J, Wilhelm-Benartzi CS (2013). Plasma antibodies to oral bacteria and risk of pancreatic cancer in a large European prospective cohort study. Gut.

[19] Sun J, Zhou M, Salazar CR, Hays R, Bedi S, Chen Y, Li Y (2017). Chronic periodontal disease, periodontal pathogen colonization, and increased risk of precancerous gastric lesions. J Periodontol.

[20] Yang Y, Cai Q, Shu XO, Steinwandel MD, Blot WJ, Zheng W, Long J (2019). Prospective study of oral microbiome and colorectal cancer risk in low-income and African American populations. Int J Cancer.

[21] Chang C, Geng F, Shi X, Li Y, Zhang X, Zhao X, Pan Y (2019). The prevalence rate of periodontal pathogens and its association with oral squamous cell carcinoma. Appl Microbiol Biotechnol.

[22] Kerdreux M, Edin S, Löwenmark T (2023). Porphyromonas gingivalis in colorectal cancer and its association to patient prognosis. J Cancer.

[23] Yuan X, Liu Y, Kong J (2017). Different frequencies of Porphyromonas gingivalis infection in cancers of the upper digestive tract. Cancer Lett.

[24] Coussens LM, Werb Z (2002). Inflammation and cancer. Nature.

[25] Wroblewski LE, Peek RM Jr, Wilson KT (2010). Helicobacter pylori and gastric cancer: factors that modulate disease risk. Clin Microbiol Rev.

[26] Kaliamoorthy S, Nagarajan M, Sethuraman V, Jayavel K, Lakshmanan V, Palla S (2022). Association of Alzheimer's disease and periodontitis - a systematic review and meta-analysis of evidence from observational studies. Med Pharm Rep.

[27] Yao Y, Shen X, Zhou M, Tang B (2021). Periodontal pathogens promote oral squamous cell carcinoma by regulating ATR and NLRP3 inflammasome. Front Oncol.

[28] Wei W, Li J, Shen X (2022). Oral microbiota from periodontitis promote oral squamous cell carcinoma development via γδ T cell activation. mSystems.

[29] Lu L, Mullins CS, Schafmayer C, Zeißig S, Linnebacher M (2021). A global assessment of recent trends in gastrointestinal cancer and lifestyle-associated risk factors. Cancer Commun (Lond).

[30] Ahn J, Chen CY, Hayes RB (2012). Oral microbiome and oral and gastrointestinal cancer risk. Cancer Causes Control.

[31] Nicolae FM, Didilescu AC, Șurlin P (2022). Subgingival periopathogens assessment and clinical periodontal evaluation of gastric cancer patients—a cross sectional pilot study. Pathogens.

[32] Sobocki BK, Basset CA, Bruhn-Olszewska B (2022). Molecular mechanisms leading from periodontal disease to cancer. Int J Mol Sci.

[33] Liu XB, Gao ZY, Sun CT, Wen H, Gao B, Li SB, Tong Q (2019). The potential role of P.gingivalis in gastrointestinal cancer: a mini review. Infect Agent Cancer.

[34] Zhou Y, Luo GH (2019). Porphyromonas gingivalis and digestive system cancers. World J Clin Cases.

[35] Meng F, Li R, Ma L (2019). Porphyromonas gingivalis promotes the motility of esophageal squamous cell carcinoma by activating NF-κB signaling pathway. Microbes Infect.

[36] Gao S, Li S, Ma Z (2016). Presence of Porphyromonas gingivalis in esophagus and its association with the clinicopathological characteristics and survival in patients with esophageal cancer. Infect Agent Cancer.

[37] Tan Q, Ma X, Yang B (2022). Periodontitis pathogen Porphyromonas gingivalis promotes pancreatic tumorigenesis via neutrophil elastase from tumor-associated neutrophils. Gut Microbes.

[38] Hiraki D, Uehara O, Kuramitsu Y (2020). P. gingivalis lipopolysaccharide stimulates the upregulated expression of the pancreatic cancer-related genes regenerating islet-derived 3 A/G in mouse pancreas. Int J Mol Sci.

[39] Mu W, Jia Y, Chen X, Li H, Wang Z, Cheng B (2020). Intracellular Porphyromonas gingivalis promotes the proliferation of colorectal cancer cells via the MAPK/ERK signaling pathway. Front Cell Infect Microbiol.

[40] Wang X, Jia Y, Wen L (2021). Porphyromonas gingivalis promotes colorectal carcinoma by activating the hematopoietic NLRP3 inflammasome. Cancer Res.

[41] Stasiewicz M, Karpiński TM (2022). The oral microbiota and its role in carcinogenesis. Semin Cancer Biol.

[42] Kylmä AK, Jouhi L, Listyarifah D (2018). Treponema denticola chymotrypsin-like protease as associated with HPV-negative oropharyngeal squamous cell carcinoma. Br J Cancer.

[43] Nieminen MT, Listyarifah D, Hagström J (2018). Treponema denticola chymotrypsin-like proteinase may contribute to orodigestive carcinogenesis through immunomodulation. Br J Cancer.

[44] Lund Håheim L, Thelle DS, Rønningen KS (2022). Low level of antibodies to the oral bacterium Tannerella forsythia predicts bladder cancers and Treponema denticola predicts colon and bladder cancers: a prospective cohort study. PLoS ONE.

